# Comparison of Narrowband Imaging with Autofluorescence Imaging for Endoscopic Visualization of Superficial Squamous Cell Carcinoma Lesions of the Esophagus

**DOI:** 10.1155/2012/507597

**Published:** 2012-10-30

**Authors:** Haruhisa Suzuki, Yutaka Saito, Ichiro Oda, Tsuyoshi Kikuchi, Shinsuke Kiriyama, Shusei Fukunaga

**Affiliations:** Endoscopy Division, National Cancer Center Hospital, 5-1-1 Tsukiji, Chuo-ku, Tokyo 104-0045, Japan

## Abstract

*Aim*. To compare narrowband imaging (NBI) and autofluorescence imaging (AFI) endoscopic visualization for identifying superficial esophageal squamous cell carcinoma (SCC). *Methods*. Twenty-four patients with superficial esophageal carcinomas diagnosed at previous hospitals were enrolled in this study. Lesions were initially detected using white-light endoscopy and then observed with both NBI and AFI. Endoscopic images documented each method, and three endoscopists experienced in esophageal imaging retrospectively reviewed respective images of histologically confirmed esophageal SCCs. Images were assessed for quality in identifying superficial SCCs and rated as excellent, fair, or poor by the three reviewers with interobserver agreement calculated using kappa (**κ**) statistics. *Results*. Thirty-one lesions histologically confirmed as superficial esophageal SCCs were detected in 24 patients. NBI images of 27 lesions (87%) were rated as excellent, three as fair, and one as poor compared to AFI images of 19 lesions (61%) rated as excellent, 10 as fair and two as poor (*P* < 0.05). Moderate interobserver agreement (*κ* = 0.42, 95% CI 0.24–0.60) resulted in NBI while fair agreement (*κ* = 0.35, 95% CI 0.18–0.51) was achieved using AFI. *Conclusion*. NBI may be more effective than AFI for visualization of esophageal SCC.

## 1. Introduction

Forty to fifty years ago, esophageal squamous cell carcinoma (SCC) was considered a devastating disease because of its aggressive clinical course and poor prognosis with five-year overall survival rates of 20–40%. The prognosis for esophageal SCC has been improving in recent years because earlier detection increases the possibility of curative treatment including esophagectomy with three-field lymph-node dissection [1, 2] and endoscopic resection [3, 4]. In particular, the prognosis of patients treated for carcinomas confined to the intraepithelium or mucosal layer of the esophagus has been excellent with five-year survival rates reportedly ranging from 85% to 100% [5, 6].

Lugol chromoendoscopy (LC) is the gold standard examination method and has been widely used in high-risk esophageal SCC populations with the number of superficial SCCs that have been detected increasing considerably [7–9]. Adverse effects such as retrosternal pain and discomfort, however, can sometimes occur because of the mucosal irritation caused by Lugol staining [10–14].

In order to detect esophageal SCC at an earlier stage without Lugol staining a need exists for the development of a new effective endoscopic method of detection. The narrowband imaging (NBI) [15–22] and autofluorescence imaging (AFI) [22–27] videoendoscope systems have recently been developed as noninvasive optical-digital methods. It has been reported that both systems have an advantage over standard white light endoscopy (WLE) so they may be useful endoscopic method for detection of early SCC lesions of the esophagus. There are limited reported data actually comparing endoscopic visualization of superficial esophageal SCC using NBI and AFI. Our aim was to compare endoscopic visualization of NBI without magnification with AFI for recognizing superficial SCC of the esophagus and assess interobserver agreement among three participating reviewers.

## 2. Materials and Methods

### 2.1. Endoscopic Imaging Systems: NBI Videoendoscope System and AFI Videoendoscope System

 We used an endoscopic imaging system that consisted of a high-resolution white-light endoscope with an optical zoom of 80x magnification (GIF-FQ260Z; Olympus Medical Systems Co., Tokyo, Japan) equipped with both NBI [15–22] and AFI [22–27] modes. Although the basic configuration is identical to that of the standard videoendoscopy system (LUCERA CV-260/CLV-260; Olympus Medical Systems), the GIF-FQ260Z allows either red, green, or blue illumination for WLE and NBI as well as an excitation/reflected light illumination combination for AFI. The light source incorporates a rotary filter designed in a double-wheel configuration with two concentric wheels including a red, green, and blue filter wheel for WLE and NBI and an AFI filter wheel.

In the NBI mode, the light source for this endoscope is equipped with narrowband filters corresponding to red (485–515 nm), green (430–460 nm), and blue (400–430 nm) light. Short wavelength light in the blue range is absorbed by hemoglobin *in vivo* enhancing the appearance of capillaries in the superficial mucosa of neoplastic areas. In the AFI mode, light emitted from a xenon lamp is directed at the rotary filter which then splits the light into excitation wavelengths of 390–470 nm and green light of 540–560 nm wavelengths. This AFI-equipped endoscope incorporates a monochrome charged couple device with a barrier filter to exclude the excitation light and capture only weak autofluorescence reflected light. A pseudocolored image is reconstructed based on the autofluorescence input signals with high-intensity autofluorescence appearing green and low-intensity autofluorescence appearing magenta. Neoplastic areas involve a thickening of the mucosal layer and increased hemoglobin, so such areas emit weaker autofluorescence compared to nonneoplastic areas. A lesion suspected of being an esophageal SCC, therefore, was defined as a demarcated area brownish in color using NBI and a purple or magenta demarcated area on a green background using AFI. This endoscopic imaging system provides endoscopic images in all three modes and makes it possible to switch to NBI or AFI endoscopy and back to WLE by pressing a single button on the endoscope handle.

### 2.2. Patients

Twenty-four consecutive patients with superficial esophageal carcinomas previously detected in other hospitals were enrolled at the National Cancer Center Hospital in Tokyo from April 2006 to September 2007. Endoscopists at the previous hospitals had used WLE as well as LC, but not NBI or AFI videoendoscopy, to detect the lesions which were all histologically confirmed as being SCCs. Two or three weeks after the initial diagnoses of esophageal SCC in the other hospitals, these 24 patients were referred to our hospital for treatment and then underwent endoscopy from one to two weeks later. Written informed consent was obtained from all patients in accordance with institutional protocol before their endoscopic examinations and treatment.

### 2.3. Endoscopic Examinations

In order to more precisely diagnose the extent of the esophageal lesions and their invasive depth for determination of the optimal method of treatment, endoscopic examinations were carried out using a videoendoscope system equipped with AFI, NBI, and NBI with magnification by a single highly experienced endoscopist (YS) familiar with both optical-digital imaging techniques. The endoscopist was provided with information received from the previous hospitals concerning the lesions including locations and number as well as various endoscopic images.

First, routine endoscopic examinations were carried out using the WLE mode to identify any abnormal mucosal areas. If an abnormal mucosal area suggesting esophageal SCC was identified, the exact location based on the distance between the upper incisor teeth and the endoscopic quadrant was recorded, and images were taken from each view. The endoscopist then examined the lesion suspected of being an esophageal SCC by switching to the NBI and AFI modes. During these examinations, images depicting the suspected esophageal lesions in the center of the endoscopic monitor were taken using both the NBI and AFI modes, and a representative selection for such lesions was then assembled of both NBI and AFI images.

In addition, NBI with magnification and LC were subsequently performed to diagnose lesions more precisely. Finally, biopsy specimens were taken from those areas suspected of being esophageal SCCs. We confirmed the lesions detected first by WLE and then by NBI, AFI, and/or LC were the same based on their exact locations as determined by measuring the distance between the upper incisor teeth and the endoscopic quadrant.

**Figure 1:**
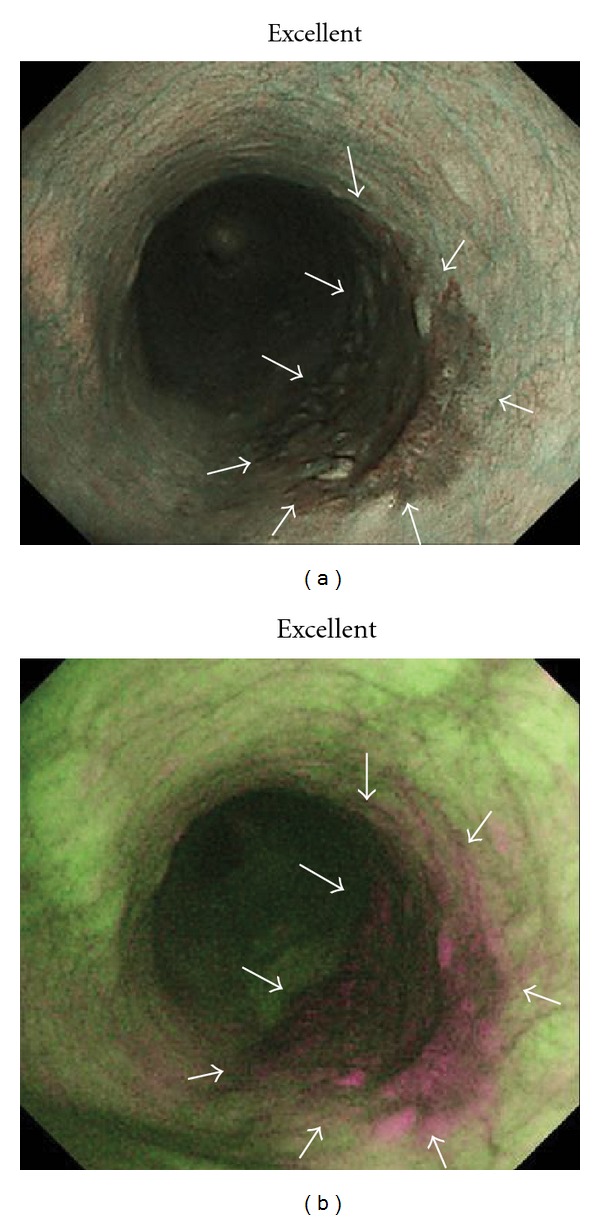
Squamous cell carcinoma (SCC) in middle esophagus (mucosal invasion: carcinoma *in situ*; depressed type; 30 mm). (a) Narrowband imaging (NBI) endoscopy clearly revealed demarcated area brownish in color with NBI image visualization quality rated as excellent for delineating entire lesion margin circumference. (b) Autofluorescence imaging (AFI) endoscopy clearly revealed demarcated area magenta in color with AFI image visualization quality rated as excellent for delineating entire lesion margin circumference.

**Figure 2:**
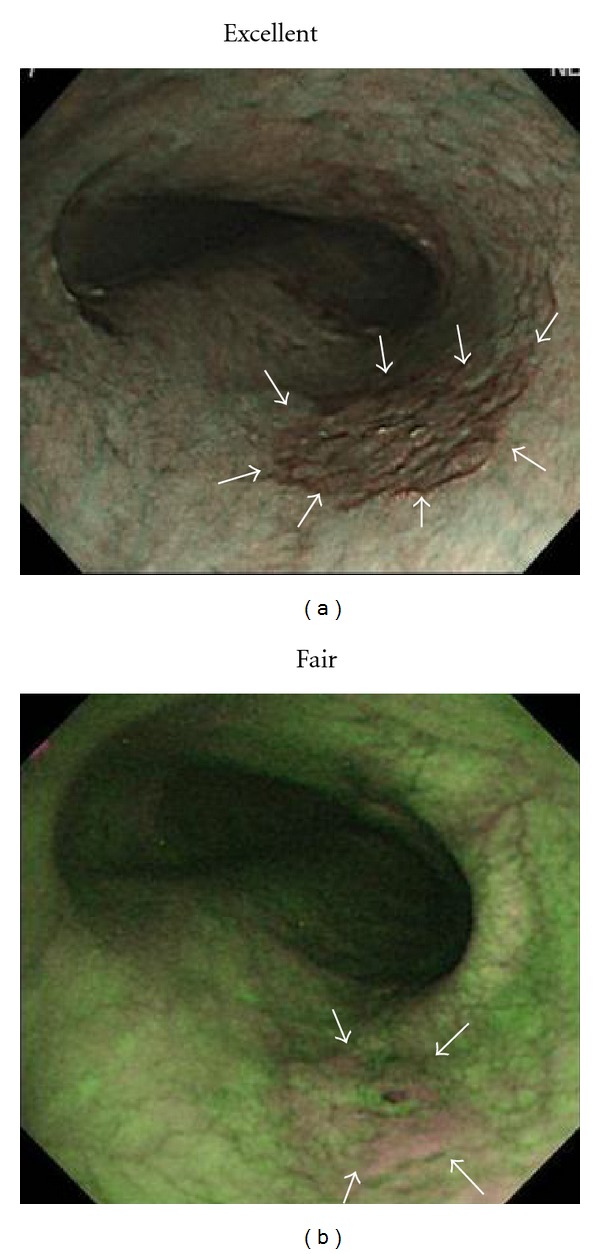
SCC in middle esophagus (mucosal invasion: carcinoma *in situ*; depressed type; 15 mm). (a) NBI endoscopy clearly revealed demarcated area brownish in color with NBI image visualization quality rated as excellent for delineating entire lesion margin circumference. (b) AFI endoscopy revealed demarcated area magenta in color with AFI image visualization quality rated as fair for delineating approximately one-half of lesion margin circumference as remaining portion of lesion margin appeared dim (white arrows).

**Figure 3:**
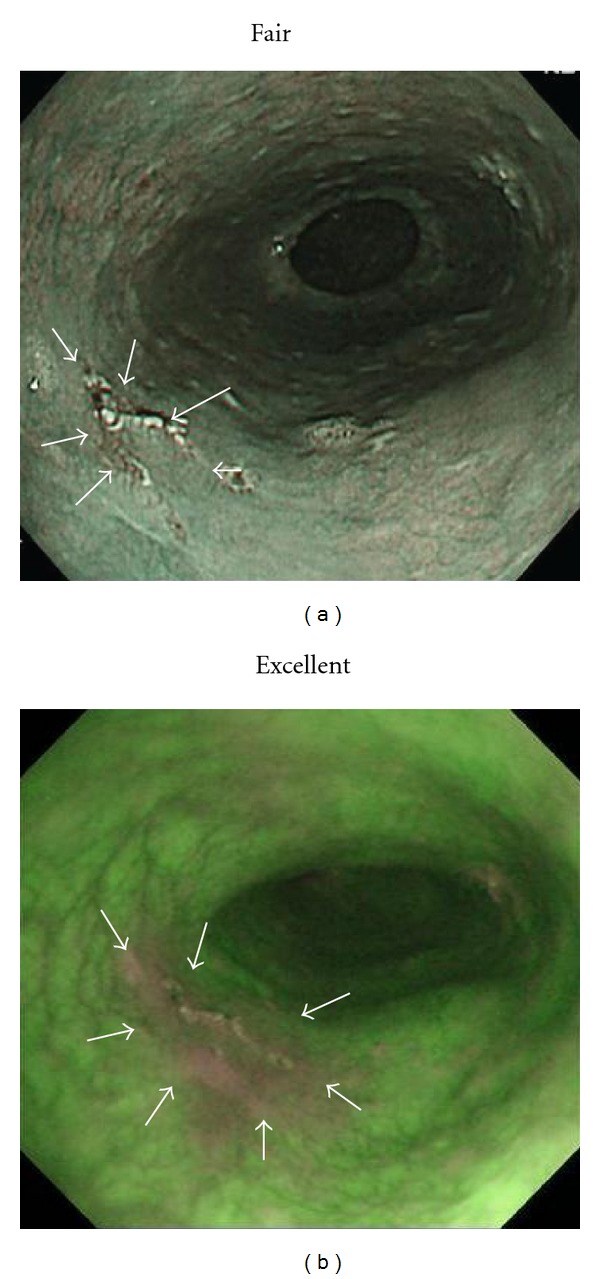
SCC in middle esophagus (mucosal invasion: carcinoma *in situ*; depressed type; 11 mm). (a) NBI endoscopy revealed demarcated area brownish in color with NBI image visualization quality rated as fair for delineating approximately one-half of lesion margin circumference as remaining portion of lesion margin appeared dim (white arrows). (b) AFI endoscopy clearly revealed demarcated area magenta in color with AFI image visualization quality rated as excellent for delineating entire lesion margin circumference.

**Figure 4:**
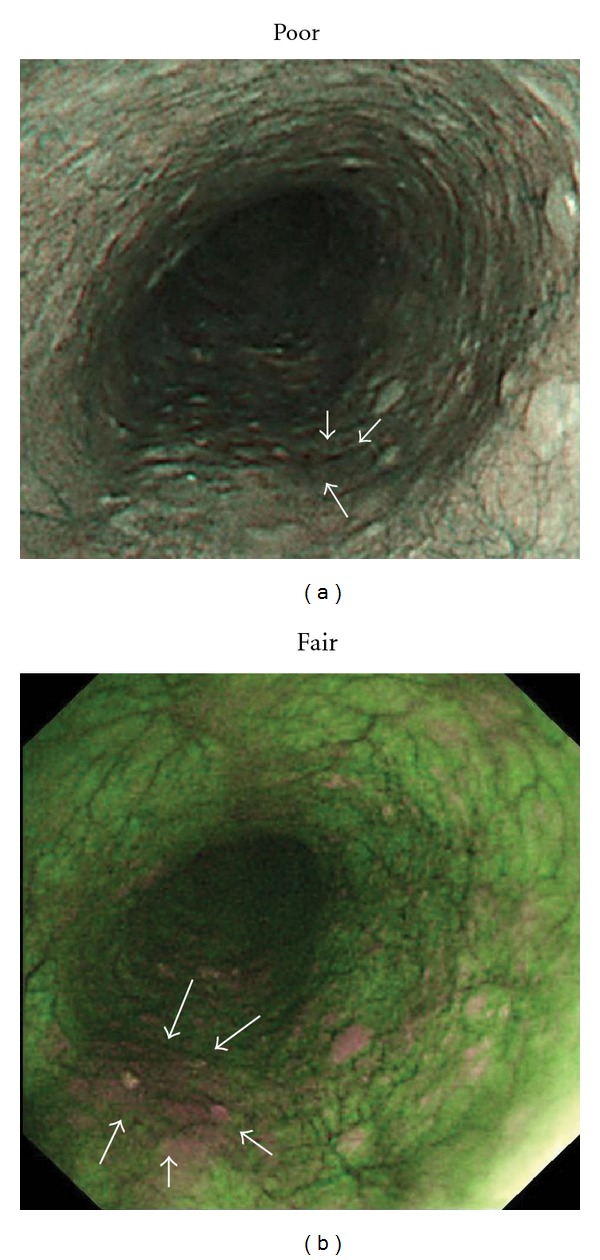
SCC in middle esophagus (mucosal invasion: proper mucosal layer; depressed type; 12 mm). (a) NBI endoscopy revealed demarcated area brownish in color with NBI image visualization quality rated as poor for delineating less than one-third of lesion margin circumference as most of lesion margin appeared dim (white arrows). (b) AFI endoscopy revealed demarcated area magenta in color with AFI image visualization quality rated as fair for delineating approximately one-half of lesion margin circumference as remaining portion of lesion margin appeared dim (white arrows).

**Figure 5:**
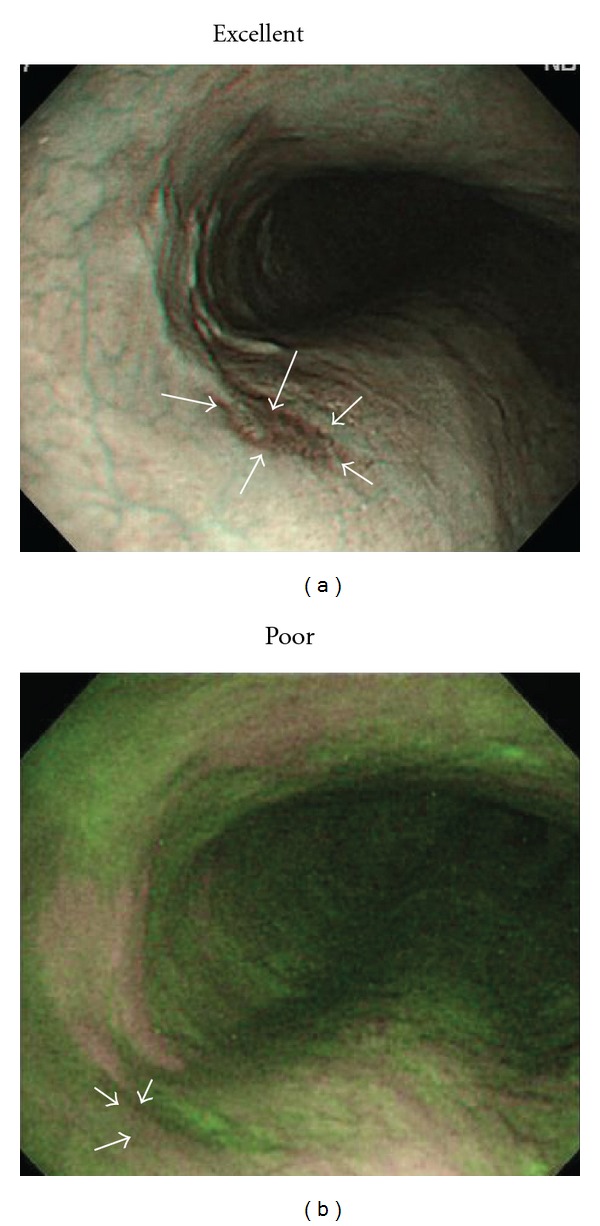
SCC in middle esophagus (mucosal invasion: carcinoma *in situ*; depressed type; 5 mm). (a) NBI endoscopy clearly revealed demarcated area brownish in color with NBI image visualization quality rated as excellent for delineating entire lesion margin circumference. (b) AFI endoscopy revealed demarcated area magenta in color with AFI image visualization quality rated as poor for delineating less than one-third of lesion margin circumference as most of lesion margin appeared dim (white arrows).

After the endoscopic examinations, three other endoscopists with extensive experience in esophageal imaging (IO, SK, and SF) retrospectively reviewed the NBI and AFI endoscopic images obtained from histologically confirmed esophageal SCCs. Each image was assessed for quality by evaluating visualization of lesion margins and rated as being excellent, fair, or poor. Assessments of image quality for each modality were performed separately to avoid any carryover effect from one endoscopic mode to the other mode. An “excellent” visualization was defined as an image in which endoscopic margins could clearly be delineated for at least two-thirds of the entire lesion circumference by NBI or AFI with the lesion then definitely diagnosed endoscopically as an esophageal SCC like LC. A “fair” visualization was defined as the image of such a lesion in which endoscopic margins could clearly be delineated for at least one-third, but less than two-thirds of the lesion circumference by NBI or AFI while the remaining portion of the lesion margin appeared dim on the image. In other words, a fair visualization was a borderline situation as to whether or not the lesion margin was sufficiently delineated in the image. A “poor” visualization was defined as the image of such a lesion in which endoscopic margins could clearly be delineated for less than one-third of the lesion circumference by NBI or AFI with most of the lesion margin appearing dim in the image. Interobserver agreement among the three reviewers was also assessed in relation to their visualization of esophageal SCCs. Representative NBI and corresponding AFI images of esophageal SCCs are shown in Figures 1, 2, 3, 4, and 5.

### 2.4. Histological Assessment and Definition of Superficial Cancer

We subsequently performed endoscopic resection on those lesions diagnosed as esophageal cancers confined to the intraepithelium or proper mucosal layer and esophagectomy on those esophageal lesions suspected of having invaded the muscularis mucosa or submucosa. Histological assessment of the endoscopically and surgically resected esophageal specimens was based on the Vienna classification [28]. Category 4 lesions under the Vienna classification are either high-grade dysplasia (4.1) or carcinoma *in situ* (4.2) while category 5 lesions are either intramucosal carcinoma (5.1) or submucosal carcinoma and beyond (5.2). Superficial esophageal cancer is defined as a lesion in which tumor invasion is limited to the intramucosal and submucosal layers corresponding to categories 4 and 5 in the Vienna classification [29].

### 2.5. Statistical Analysis

In order to compare the image quality of esophageal SCCs using NBI without magnification with AFI, McNemar's Test was used for statistical analysis with the standard computer software statistical package, SPSS for Windows (SPSS, Release 6.0; SPSS Inc., Chicago, Illinois, USA). A *P*  value < 0.05 was considered significant. Interobserver agreement among the three reviewers was calculated using kappa (*κ*) statistics based on Landis and Koch criteria with *κ*-values interpreted as being poor (<0), slight (0.0–0.20), fair (0.21–0.40), moderate (0.41–0.6), substantial (0.61–0.8), and almost perfect to perfect (0.81–1.00) agreement.

## 3. Results

We identified a total of 31 superficial esophageal SCC lesions in the 24 patients. These lesions were characterized and diagnosed according to their respective location in the esophagus (upper, middle, and lower: 4, 17, and 10); esophageal lumen circumferential ratio (<1/2; 1/2 or more/<3/4; 3/4 or more: 18, 10, and 3); macroscopic type (depressed and elevated: 29 and 2); lesion size (≤20 mm and >20 mm: 15 and 16); depth of invasion (mucosal and submucosal: 27 and 4) (Table 1). Endoscopic resection was performed on 20 patients with 26 lesions, and esophagectomy was carried out on the remaining four patients who had five lesions between them.

**Figure 6:**
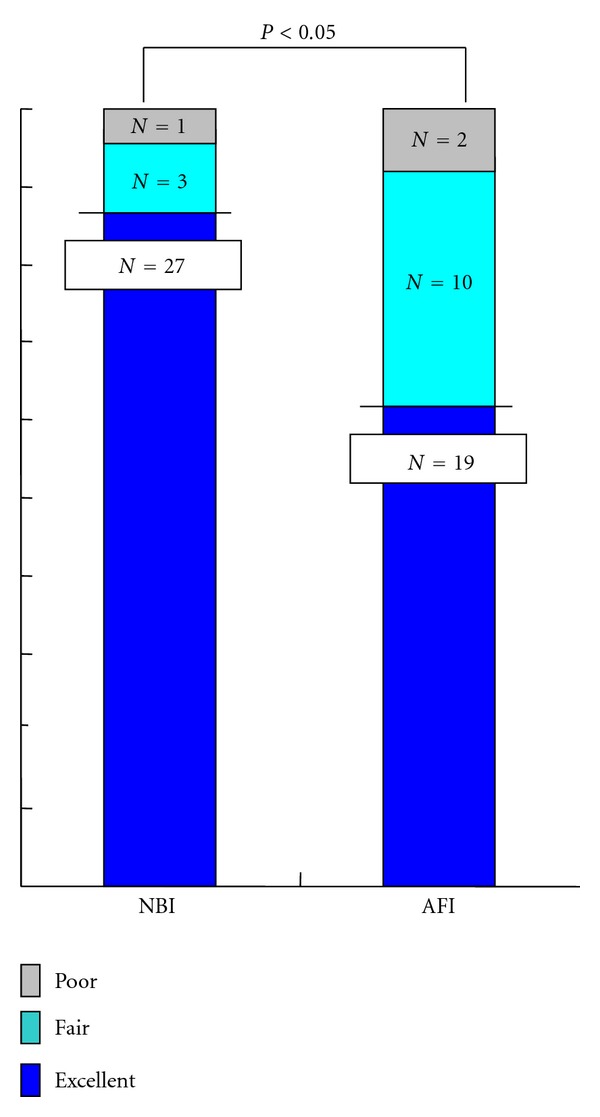
Comparative visualization of superficial esophageal SCCs by NBI and AFI.

Using NBI, 27 lesions (87%) were rated as excellent, three as fair, and one as poor whereas 19 (61%) lesions were rated as excellent, 10 as fair, and two as poor with AFI (*P* < 0.05) (Figure 6). In terms of interobserver agreement among the three reviewers on the visualization of superficial esophageal SCCs, moderate agreement (*κ* = 0.42, 95% CI 0.24–0.60) was achieved using NBI and fair agreement (*κ* = 0.35, 95% CI 0.18–0.51) with AFI.

**Figure 7:**
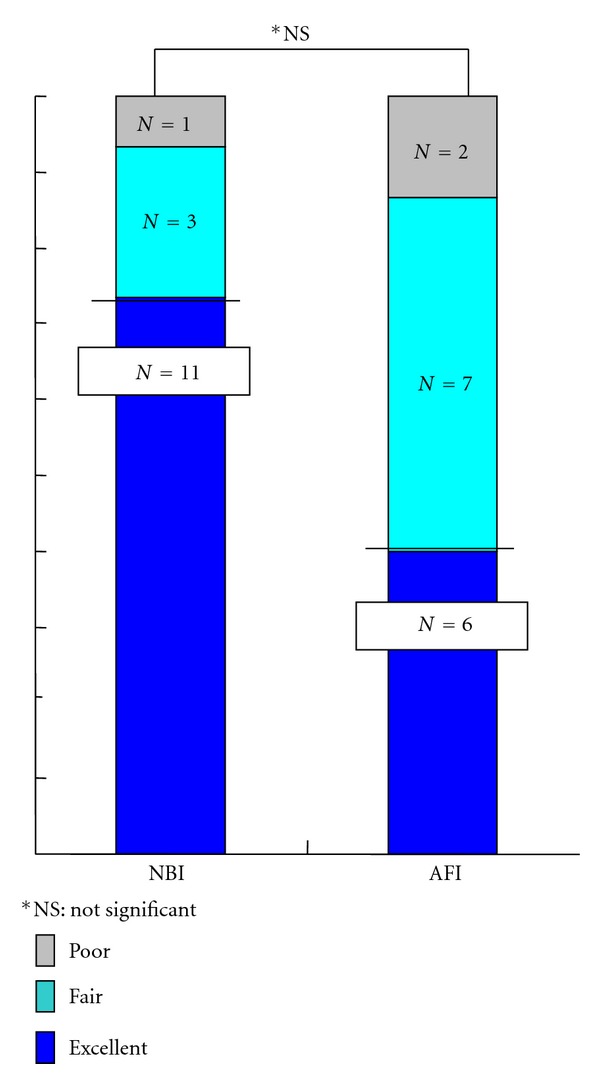
Comparative visualization of superficial esophageal SCCs consisting of depressed type mucosal lesions ≤20 mm in size by NBI and AFI.

As for 15 depressed lesions limited to the mucosa and ≤20 mm in size that were particularly difficult to visualize using WLE, 11 lesions (73%) were rated as excellent, three as fair, and one as poor with NBI whereas six (40%) lesions were rated as excellent, seven as fair, and two as poor using AFI. The difference between the two imaging systems, however, was not statistically significant for such depressed lesions (Figure 7).

## 4. Discussion

Based on the results of our study, the NBI videoendoscope system visualized superficial esophageal SCCs better compared to the AFI system. This result suggests, therefore, that the NBI system may be more useful for the visualization of esophageal SCC compared to AFI.

The early detection of superficial esophageal SCC by conventional WLE continues to be difficult [5, 7] because there are so few morphological changes, but LC improves endoscopic visualization and frequently makes it possible to detect esophageal SCC at an early stage [7–9]. In order to improve detection of early stage esophageal SCC, therefore, widespread use of Lugol staining has been recommended in high-risk populations such as heavy drinkers and heavy smokers. Unfortunately, Lugol staining often causes mucosal irritation during examinations leading to retrosternal pain and discomfort [10–14] although thorough rinsing with thiosulfate solution at the conclusion of the examination can reduce such irritation. Consequently, the development of a new, noninvasive diagnostic modality has become highly desirable in recent years for detecting esophageal SCC. Under such circumstances, both NBI and AFI were developed as noninvasive optical-digital methods.

The AFI videoendoscope system can distinguish neoplastic from nonneoplastic tissue [22–27], and there have been recent reports indicating the AFI system had an advantage over standard WLE in the detection of early esophageal cancers with AFI image quality being acceptable for the purpose of such detection [25, 26]. The NBI system is another novel, noninvasive optical-digital imaging method that has shown promising results in the detection of esophageal and pharyngeal SCC [15–22]. In addition, magnification endoscopy conducted with NBI can reveal morphological changes in the capillary vessels of such SCCs so as to distinguish between neoplastic lesions and inflammatory conditions and be useful in predicting histological depth of invasion [15–19]. Both the NBI [15–22] and AFI [22–26] systems could play an important role in the future detection of such cancer because each system has been shown to improve the endoscopic visualization of esophageal SCCs without any of the disadvantages associated with LC.

In this study, NBI provided superior visualization of esophageal SCCs compared to AFI despite the fact that NBI was used without magnification. It seems reasonable to conclude from our results, therefore, that NBI has greater potential for enhanced endoscopic visualization of esophageal SCC in comparison to AFI. Our study, however, had several limitations. First, we did not conduct a comparison of NBI and AFI in the detection of esophageal SCCs. This was an uncontrolled pilot trial comparing NBI to AFI for the visualization of such lesions initially detected by WLC in a relatively small number of patients without nonneoplastic lesions being included in our limited study with interobserver agreement based on only three reviewers. In addition, problems had previously been reported in the visualization of some lesions and in distinguishing neoplastic lesions from inflammatory changes using AFI because the AFI videoendoscope system can produce false-positive findings attributable to inflammation due to resolution limitations [27]. It has also been reported that NBI can produce false-positive findings caused by benign pathologies such as inflammatory changes [30]. We did not assess the ability of NBI and AFI to detect superficial esophageal SCCs, however, so we did not describe the clinicopathological features of lesions with false-positive findings for each modality. Consequently, a prospective randomized controlled trial involving a larger number of patients with not only esophageal SCCs but also nonneoplastic lesions should be conducted in the future to compare the esophageal SCC detection capabilities of both the NBI and AFI utilizing a new videoendoscope system with improved image resolution. In addition, we should clarify the clinicopathological features of those lesions with false-positive findings for each modality in order to improve diagnostic accuracy of each modality.

In conclusion, the results of this study indicated that the NBI videoendoscope system was more effective for the visualization of esophageal SCC because NBI provided better visualization of such lesions compared to AFI.

## Figures and Tables

**Table 1:** Clinicopathological features of superficial esophageal squamous cell carcinomas and visualization by narrow-band imaging (NBI) and autofluorescence imaging (AFI).

Case	Lesion	Location	Circumference of esophageal lumen	Macroscopic type	Size, mm	Depth of invasion (Vienna classification)	Treatment	Visualization	
								NBI	AFI
1	1	Lower	<1/2	Depressed	15	Mucosal (4.2)	ER*	Fair	Fair
2	2	Middle	1/2≤ <3/4	Depressed	53	Mucosal (5.1)	ER	Excellent	Excellent
3	3	Middle	<1/2	Depressed	5	Mucosal (4.2)	ER	Excellent	Poor
	4	Middle	<1/2	Depressed	30	Mucosal (5.1)	ER	Excellent	Excellent
	5	Middle	<1/2	Depressed	15	Mucosal (4.2)	ER	Excellent	Fair
4	6	Upper	1/2≤ <3/4	Depressed	35	Mucosal (5.1)	ER	Excellent	Excellent
5	7	Middle	<1/2	Depressed	10	Mucosal (5.1)	ER	Excellent	Fair
6	8	Middle	<1/2	Depressed	3	Mucosal (4.2)	ER	Excellent	Fair
7	9	Middle	<1/2	Depressed	23	Mucosal (5.1)	ER	Excellent	Fair
8	10	Lower	1/2≤ <3/4	Depressed	43	Mucosal (5.1)	ER	Excellent	Excellent
9	11	Middle	<1/2	Depressed	11	Mucosal (4.2)	ER	Fair	Excellent
10	12	Lower	3/4≤	Depressed	60	Mucosal (5.1)	Surgery	Excellent	Fair
11	13	Lower	1/2≤ <3/4	Depressed	25	Mucosal (5.1)	ER	Excellent	Excellent
12	14	Middle	1/2≤ <3/4	Elevated	30	Submucosal (5.2)	Surgery	Excellent	Excellent
	15	Middle	<1/2	Depressed	10	Mucosal (5.1)	Surgery	Excellent	Excellent
13	16	Middle	1/2≤ <3/4	Depressed	64	Mucosal (5.1)	ER	Excellent	Excellent
14	17	Middle	<1/2	Depressed	18	Mucosal (5.1)	ER	Excellent	Poor
	18	Lower	<1/2	Depressed	15	Mucosal (4.2)	ER	Excellent	Fair
	19	Middle	<1/2	Depressed	12	Mucosal (5.1)	ER	Poor	Fair
	20	Upper	<1/2	Depressed	10	Mucosal (4.2)	ER	Excellent	Fair
15	21	Lower	1/2≤ <3/4	Depressed	50	Sub mucosal (5.2)	ER	Excellent	Excellent
16	22	Upper	1/2≤ <3/4	Depressed	18	Mucosal (5.1)	ER	Excellent	Excellent
17	23	Middle	1/2≤ <3/4	Depressed	30	Mucosal (5.1)	Surgery	Excellent	Excellent
18	24	Lower	<1/2	Depressed	20	Mucosal (5.1)	ER	Fair	Excellent
19	25	Lower	1/2≤ <3/4	Depressed	26	Mucosal (4.2)	ER	Excellent	Excellent
20	26	Middle	3/4≤	Elevated	100	Sub mucosal (5.2)	Surgery	Excellent	Fair
21	27	Middle	<1/2	Depressed	15	Mucosal (5.1)	ER	Excellent	Excellent
22	28	Lower	<1/2	Depressed	30	Mucosal (5.1)	ER	Excellent	Excellent
	29	Upper	<1/2	Depressed	10	Mucosal (4.2)	ER	Excellent	Excellent
23	30	Lower	3/4≤	Depressed	40	Sub mucosal (5.2)	ER	Excellent	Excellent
24	31	Middle	<1/2	Depressed	30	Mucosal (4.2)	ER	Excellent	Excellent

^∗^ ER:  endoscopic resection.
